# Open, hybrid, minimally invasive, and robotic-assisted transthoracic esophagectomy for cancer: a network meta-analysis of randomized trials

**DOI:** 10.1097/JS9.0000000000002985

**Published:** 2025-07-07

**Authors:** Alberto Aiolfi, Francesco Cammarata, Gianluca Bonitta, Davide Bona, Luigi Bonavina

**Affiliations:** a Division of General Surgery, Department of Biomedical Science for Health, I.R.C.C.S. Ospedale Galeazzi – Sant’Ambrogio, University of Milan, Milan, Italy; b Department of Biomedical Sciences for Health, Division of General and Foregut Surgery, IRCCS Policlinico San Donato, University of Milan, Milan, Italy

**Keywords:** esophageal cancer, hybrid esophagectomy, minimally invasive esophagectomy, open esophagectomy, robotic-assisted esophagectomy

## Abstract

**Background:**

The surgical approach to esophageal cancer is evolving. Open (OE) and hybrid esophagectomy (HE) have been standard treatments for years but minimally invasive (MIE) and robotic-assisted esophagectomy (RAMIE) have recently emerged with promising perspectives.

**Purpose:**

Compare short-term outcomes among different techniques for transthoracic esophagectomy.

**Methods:**

Systematic review and random effect Frequentist network meta-analysis. Included were randomized controlled trials (RCTs) reporting short-term outcomes for transthoracic OE, HE, MIE, and RAMIE in adult patients undergoing esophagectomy for cancer. Primary outcomes were anastomotic leak, pulmonary complications (PCs), and in-hospital mortality. Risk ratio (RR), standardized mean difference, and 95% confidence intervals (CIs) were used as pooled effect size measures. PROSPERO (CRD42025645818).

**Results:**

Eight RCTs (1776 patients) were included. Overall, 493 patients (27.8%) underwent OE, 494 (27.9%) HE, 447 (25.3%) MIE, and 338 (19.2%) RAMIE. Adenocarcinoma was diagnosed 65.8% of patients while neoadjuvant therapy was completed in 64.1%. Ivor Lewis or McKeown esophagectomy was performed in 43.3% and 56.7% of patients, respectively. MIE and RAMIE were associated with a reduced risk of PCs compared to OE (RR = 0.46; 95% CI 0.29–0.71 and RR = 0.48; 95% CI 0.33–0.71) and HE (RR = 0.54; 95% CI 0.34–0.86 and RR = 0.57; 95% CI 0.37–0.87). Additionally, MIE and RAMIE showed significantly reduced intraoperative blood loss and hospital length of stay compared to OE and HE. Among surgical approaches, anastomotic leak, in-hospital mortality, together with the other perioperative and oncological outcomes were equivalent.

**Conclusions:**

MIE and RAMIE were associated with significantly reduced postoperative PCs, intraoperative blood loss and shorter hospital stays compared to OE and HE. MIE and RAMIE showed equivalent perioperative outcomes and oncological radicality.

## Introduction

Esophageal cancer ranks as the eighth most frequently diagnosed cancer globally and is the sixth leading cause of cancer-related fatalities^[1,2]^. Surgery is the cornerstone of treatment, usually associated with perioperative chemotherapy or included in trimodal chemoradiation protocols^[3–5]^. However, esophagectomy is a highly invasive procedure, resulting in significant morbidity and mortality^[6,7]^. In recent years, the surgical techniques for transthoracic esophagectomy have evolved, particularly with the introduction of minimally invasive esophagectomy (MIE)^[8–11]^. Luketich *et al*^[12]^ first described the totally MIE approach, incorporating both thoracoscopic and laparoscopic phases along with a cervical incision for anastomosis. Since then, MIE has gained increasing acceptance, with studies demonstrating its feasibility, safety, and promising results^[13]^. There have been some concerns regarding the protracted learning curve, the potential for higher morbidity, and the need for a high hospital volume to achieve outcomes comparable to those of the open technique^[14]^. The growth of innovative robotic platforms has since opened new perspectives, beginning with the first robot-assisted MIE (RAMIE)^[15–17]^. The improved ergonomics, high-definition 3-D visualization, and increased motion allowed by instruments with multiple degrees of freedom have contributed to the progressive global spread of RAMIE^[18–20]^.

Previous observational studies indicate that MIE and RAMIE are equally safe with shorter hospital stays, and comparable oncologic outcomes when compared to open (OE) and hybrid esophagectomy (HE)^[15–18,21,22]^. Additionally, previous meta-analyses with simple pairwise comparisons concluded that MIE and RAMIE show improved short-term outcomes compared to OE and HE^[23–26]^. However, a comprehensive network meta-analysis simultaneously comparing these four major approaches to transthoracic esophagectomy in a single analysis is lacking. A prior network analysis aimed to address this gap, but the quantitative synthesis incorporated studies up to 2021 and was limited by overlapping patient data^[27]^.

This systematic review aims to provide thorough and updated evidence on the perioperative and oncologic outcomes for OE, HE, MIE, and RAMIE within the context of randomized controlled trials (RCTs). In addition, it intends to compare HE to RAMIE, which have not been directly compared to date.

## Materials and methods

A systematic review was performed according to the guidelines from the preferred reporting items for systematic reviews and network meta-analyses^[28]^ and the Assessing the Methodological Quality of Systematic Reviews^[28,29]^. No artificial intelligence tools were used in the research and manuscript development^[30]^. Institutional review board approval was not required. MEDLINE, Scopus, Web of Science, Cochrane Central Library, and ClinicalTrials.gov were used. The last date of search was the February 2, 2025. A combination of the following MeSH terms (Medical Subject Headings) was used (“esophageal cancer” [tiab], OR esophageal cancer” [tiab] AND (“open esophagectomy” [tiab], OR “open esoph*” [tiab]) AND (“hybrid esophagectomy” [tiab], OR “hybrid esoph*” [tiab]); (“minimally invasive esophagectomy” [tiab], OR “minimally invasive esoph*” [tiab]) AND (“robotic esophagectomy” [tiab], OR “robotic esop*” [tiab]). The comprehensive search strategy is defined in the Appendix 1. Three authors (AA, FC and GB) evaluated the title of each study, and suitable abstracts were extracted. The search was completed by consulting the references of each article. The study protocol was registered at the International prospective register of systematic reviews (PROSPERO registration number: CRD42025645818).

### Eligibility criteria

Inclusion criteria: (a) RCTs comparing outcomes for transthoracic OE, HE, MIE, and RAMIE in adult patients (>18 years) undergoing elective esophagectomy for cancer (b) English written, (c) when two or more studies were published using the same dataset, we included the study with the longest follow-up period or the largest sample size, (d) for duplicate studies, we only included the study with the complete dataset for quantitative analysis. Exclusion criteria: (a) non-English articles, (b) no clear outcome distinction between different surgical techniques, (c) studies reporting data for transhiatal esophagectomy, (d) studies including less than five patients for each treatment arm.

**HIGHLIGHTS**

- This network meta-analysis of 8 randomized controlled trials including 1776 patients indicated that minimally invasive (MIE) and robotic-assisted esophagectomy (RAMIE) were associated with significantly reduced postoperative pulmonary complications, intraoperative blood loss and shorter hospital stays compared to open (OE) and hybrid esophagectomy.
- Operative time, anastomotic leak, gastric conduit necrosis, chylothorax, vocal cord paralysis, surgical site infection, reoperation, severe postoperative complications, R0, total number of retrieved lymph nodes, and in hospital mortality were comparable among the four treatments.
- MIE and RAMIE showed equivalent perioperative outcomes and surgical radicality.

### Data extraction

The following variables were collected: authors, year of publication, country, RCTs design, number of patients, age, sex, body mass index (BMI), American Society of Anesthesiologists (ASA) physical status, comorbid conditions, surgical indications, tumor characteristics, histological type, tumor location, cancer stage, use of neoadjuvant chemoradiation therapy, and postoperative outcomes. All data were computed independently by three investigators (AA,FC,GB) and compared at the end of the review process. Two senior authors (AA and FC) clarified discrepancies.

### Outcomes

Primary outcomes were postoperative anastomotic leak, pulmonary complications’ (PCs), and in-hospital mortality defined as death from any cause before discharge. Secondary outcomes were operative time (OT) (minutes) calculated from skin to skin, estimated intraoperative blood loss (ml), conversion to open surgery, intensive care unit (ICU) length of stay (days), ICU readmission, gastric conduit necrosis (GCN), reintubation, surgical site infection (SSI), chylothorax, vocal cord paralysis confirmed by laryngoscopy at 6 weeks, return to operative room, severe postoperative complications defined as Clavien–Dindo > grade 2^[31,32]^, hospital length of stay (days), hospital readmission after discharge, total number of retrieved lymph nodes, tumor free resection margins (R0) defined as >1 mm from the resection margin, 30-day mortality, and 90-day mortality. The definition of secondary outcomes is reported in Supplemental Digital Content Table 1, available at: http://links.lww.com/JS9/E620^[32–34]^. The time frame for both primary and secondary outcomes was established as until the patient’s discharge unless stated otherwise. OE refers to the surgical removal of the esophagus along with lymph node retrieval through a combination of midline laparotomy and right thoracotomy. HE is defined as esophagectomy performed via laparoscopy and right thoracotomy. MIE consists of esophageal resection through laparoscopy and right thoracoscopy. RAMIE is defined as esophagectomy carried out entirely via robotic assistance or via laparoscopy and right robotic thoracoscopy.

### Quality assessment

Three authors (AA, FC, GB) independently assessed the methodological quality of the selected trials by using the Cochrane risk of bias tool II^[35]^. This tool evaluates five domains: (1) bias of randomization; (2) allocation concealment; (3) bias due to missing outcomes; (4) bias in the measurements of outcomes; and (5) bias in selection of the reported results. Thus, each RCT was graded as having low, moderate, or high risk of bias. Disagreements were solved by discussion.

### Statistical analysis

We performed a random effect Frequentist network meta-analysis^[36]^. The random-effects model was preferred over a fixed effect model to appropriately allocate variance within the statistical model while a Frequentist perspective was preferred because the vague and “non-informative” prior distribution. Raw data were extracted using 2 × 2 tables for each outcome measured. For studies that reported data as median and range, a technique described by Hozo *et al*^[37]^ was used to calculate an estimate of the mean and standard deviation. In particular, for studies with smaller sample sizes (*n* < 25), the median was treated as a suitable approximation of the mean. Conversely, for studies with larger samples (*n* > 25), the mean was computed using the formula (a + 2 m + b)/4, where “a” and “b” represent the lower and upper limits of the range, and “m” denotes the median. For dichotomous variables, the risk ratio (RR) was chosen as the effect size. For continuous variables, standardized mean difference was chosen as the effect size. Generalized DerSimonian-Larid^[38]^ estimator was used to estimate the between-study variance, assumed as common for each pairwise treatment comparison. A generalized *I*^2^ was adopted to define heterogeneity as follows: low (< 25%), moderate (25%–75%), or high (> 75%)^[39]^. To ensure transitivity, we structured the eligibility criteria for the included studies so that the trials differ principally in the interventions being tested. To evaluate transitivity, we created descriptive statistics and compared the baseline characteristics’ distributions across the studies and treatment comparisons^[40]^. Because the presence of open loop in the network comparisons, the formal assessment of local inconsistency was not performed as per statistical rules. The treatment ranking probability was estimated with the cumulative ranking curve. The network geometry was appraised, and the confidence of outcomes estimates was assessed with Confidence in Network Meta-Analysis (CINeMA) instrument^[41]^. Two-sided *P*-values were considered statistically significant when less than 0.05, and the confidence intervals (CI) were computed at 95%. Where necessary we estimated standard deviation from range^[37,42]^. All analyses and graphs were carried out using R-CRAN statistical software with *netmeta* package^[43]^.

## Results

### Literature search

**Figure 1.**
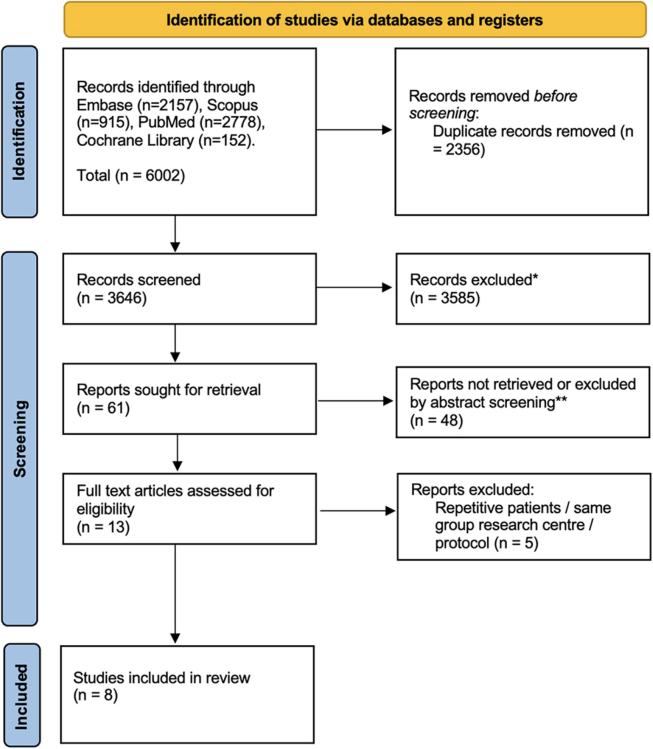
The Preferred Reporting Items for Systematic Reviews and network meta-analyses checklist (PRISMA-NMA) diagram. *Records excluded such as conference abstracts, book chapters, conference posters, unrelated topics, language issues and then filtered maintaining only clinical trials. **No direct comparison for different transthoracic esophagectomy approaches, transhiatal esophagectomy and colonic interposition.

The PRISMA flowchart is reported in Figure 1. Overall, 6002 publications were identified. After duplicates removal, 3646 titles were screened. Overall, 61 abstracts were reviewed while 13 articles were found possibly relevant for full-text assessment. After full text evaluation, 10 studies derived from 8 RCTs meet the inclusion/exclusion criteria and were included in the quantitative synthesis^[22,44–52]^.

### Risk of bias assessment

The quality of included RCTs is reported in Supplemental Digital Content Table 2, available at: http://links.lww.com/JS9/E621. Five of the RCTs had a multicenter design^[44–47,50]^. Randomization methods were specified in seven RCTs^[22,44–47,49,50]^, while details on the blinding of perioperative outcomes were provided in three studies^[22,45,47]^. Power analysis was clarified in seven RCTs^[22,44–47,49,50]^; the MIOMIE trial was considered underpowered and was halted early due to a high leak rate^[49]^. The analyses were performed for both intention-to-treat and/or per-protocol purposes. Six RCTs provide specific data regarding the operating surgeon proficiency whereas four implemented surgical quality control measures, including intraoperative images, videos, checklists, and visits from the principal investigator, to assess the quality of surgery and adherence to established protocols^[22,44–47,50]^. Five trials were deemed to have a low risk of bias^[22,45–47,50]^, two were assessed as intermediate risk^[44,49]^, and one was rated as having a high risk of bias^[52]^ (Supplemental Digital Content Figure 1A and B, available at: http://links.lww.com/JS9/E619).

### Systematic review

Overall, 1772 patients were included (Table 1). Of those, 493 patients (27.8%) underwent OE, 494 (27.9%) HE, 447 (25.3%) HE, and 338 (19.2%) RAMIE. The age of the patient population ranged from 34 to 78 years, the BMI ranged from 16 to 41 kg/m^2^ and the majority (61.6%) were males. Overall, 23.6% of patients were categorized as ASA score >2. The patient performance status was defined in three RCTs while the Charlson comorbidity index and the POSSUM score were defined in one study, respectively. Tumor histology was specified in six RCTs (1172 patients): adenocarcinoma and squamous cell carcinoma were diagnosed in 65.8% and 33.5% of patients, respectively. Tumor grading was reported in three RCTs. Tumor location was reported in all included studies and divided into upper (4.5%), middle (32.6%), and lower esophagus/gastroesophageal junction (62.9%). Pathological tumor staging and TNM according to the 6th, 7th, and 8th edition of the American Joint Committee on Cancer was detailed in all RCTs (Stage 0: 4.9%, Stage I–II: 54.6%; Stage III: 34.4%, and Stage IV: 6.1%). Neoadjuvant chemotherapy or chemoradiation therapy were completed in 64.1% of patients with heterogeneous strategies (i.e. protocols, regimens, dosages, radiation fractioning, etc.).

**Table 1:** Demographic, clinical, and operative data for patients undergoing transthoracic open (OE), hybrid (HE), minimally invasive (MIE), and robotic-assisted esophagectomy (RAMIE)

Author, year, country,	Study period	No. centers	Surgical procedure	No. pts	Age (yrs)	Gender (male)	BMI (kg/m^2^)	ASA >3	Neo	Tumor location (U/M/L or GEJ)	Histology (ADC/SCC/other)	Pathologic Stage I/II/III/IV (no residual tumor)	TNM classification		Type of resection
Trial name															
Biere *et al*, 2012 (Netherlands, Spain, Italy), TIME trial^[49,50]^	2009–2011	5	OE	56	62 (42–75)	46	24 ± 3.7	9	56	3/22/31	36/19/1	4/22/14/5 (7)	nr		IL and McKeown

			MIE	59	62 (34–75)	43	25 ± 3.6	15	59	1/26/32	35/24/0	4/26/11/4 (10)	nr		IL and McKeown

Guo *et al*, 2013, China, nr^[51]^	2006–2008	1	HE	110	60.8 ± 12.4	72	nr	nr	nr	7/76/27	nr	nr	T1-2N0M0 31		McKeown
													T3N0M0 5		
													T2-3N1M0 74		
			MIE	111	57.3 ± 11.8	68	nr	nr	nr	13/78/20	nr	nr	T1-2N0M0 24		McKeown

													T3N0M0 7		
													T2-3N1M0 80		
Paireder *et al*, 2018, Austria, MIOMIE^[48]^	2010–2012	1	OE	12	62 (49–77)	10	27 (17–35)	nr	7	0/1/11	11/1/0	nr	T0: 2	N0: 7	IL
													T1: 4T2: 2T3: 3 T4a: 1	N1: 3	
														N2: 2N3: 0	
			HE	14	64 (40–75)	100	24 (18–41)	nr	9	0/4/10	10/4/0	nr	T0: 1T1: 4T2: 2T3: 6T4a: 1	N0: 7N1: 5N2: 0N3: 2	IL
Mariette *et al*, 2019, France, MIRO^[46,47]^	2009–2012	13	OE	104	62 (41–78)	87	25 (18–35)	12	75	1/31/72	66/38/0	25/31/35/0 (0)	nr		IL
												Missing 13			

			HE	103	59 (23–75)	88	25 (16–37)	17	77	0/32/71	57/46/0	20/33/37/0 (3)	nr		IL

												Missing 10			
Van der Sluis *et al*, 2019, Netherlands, ROBOT^[22]^	2012–2016	1	RAMIE	54	64 ± 8.9	46	26 ± 4.4	6	48	1/5/48	41/13/0	4/16/34/0	nr		McKeown
			OE	55	65 ± 8.2	42	25.5 ± 4.7	10	48	0/8/47	43/12/0	4/21/30	nr		McKeown
Yang *et al*, 2022, China, RAMIE^[45]^	2017–2019	6	RAMIE	181	65 (43–75)	156	23.1 ± 2.8	nr	39	19/83/79	nr	28/94/57/2	nr		McKeown

			MIE	177	63 (42–75)	150	23 ± 3.1	nr	37	18/88/71	nr	22/93/62/0	nr		McKeown
Chao YK *et al*, 2024, China, REVATE^[43]^	2018–2022	3	RAMIE	103	60 ± 8.4	96	23 ± 3.4	41	53	11/45/47	0/103/0	40/27/26/10	nr		McKeown
			MIE	100	61 ± 7.7	88	23 ± 3.2	40	47	4/41/55	0/100/0	40/23/31/6	nr		McKeown
ROMIO 2024, UK, ROMIO^[44]^	2016–2019	8	OE	266	66 ± 9	227	27 ± 4	nr	221	1/8/246	237/25/3	I/II: 106	nr		IL
												III: 80			
												IV: 33 (23)			

			HE	267	67 ± 9	225	27 ± 4	nr	219	0/25/237	235/26/5	I/II: 109	nr		IL
												III: 82			
												IV: 28 (29)			

Data are reported as median (±standard deviation) or median (range).

ADC, adenocarcinoma; BMI, body mass index; GEJ, gastroesophageal junction; IL, Ivor Lewis esophagectomy; L, lower esophagus; M, middle esophagus; Neo, neoadjuvant treatment; nr, not reported; SCC, squamous cell carcinoma; U, upper esophagus; yrs, years.

Ivor Lewis or McKeown esophagectomy with intrathoracic or cervical anastomosis was performed in 43.3% and 56.7% of patients, respectively depending on operating surgeon preference, tumor location, and histology. The technique for esophagogastric anastomosis (i.e. hand-sewn, circular stapled or linear stapled) differ depending on operating surgeon preference. The extent of lymphadenectomy (two-field vs. extended two-field) varied depending on surgeon expertise and tumor clinical stage/location. Pyloroplasty, pyloromyotomy, or no drainage were optional; placement of jejunostomy or feeding nasojejunal tube were at surgeon discretion. Placement of thoracic/abdominal drain and use of hiatal approximation sutures to minimize diaphragmatic herniation was at surgeon discretion. Data on thoracic duct ligation or resection were puzzled. Intraoperative ventilation techniques, modalities (single lung vs. dual lung vs. bronchial blocker utilization) and ventilation pressures were not uniformly defined. Similarly, sparse or no data were available on epidural catheter utilization and intraoperative utilization of fluids/vasoconstrictors.

### Meta-analysis – primary outcomes

**Figure 2.**
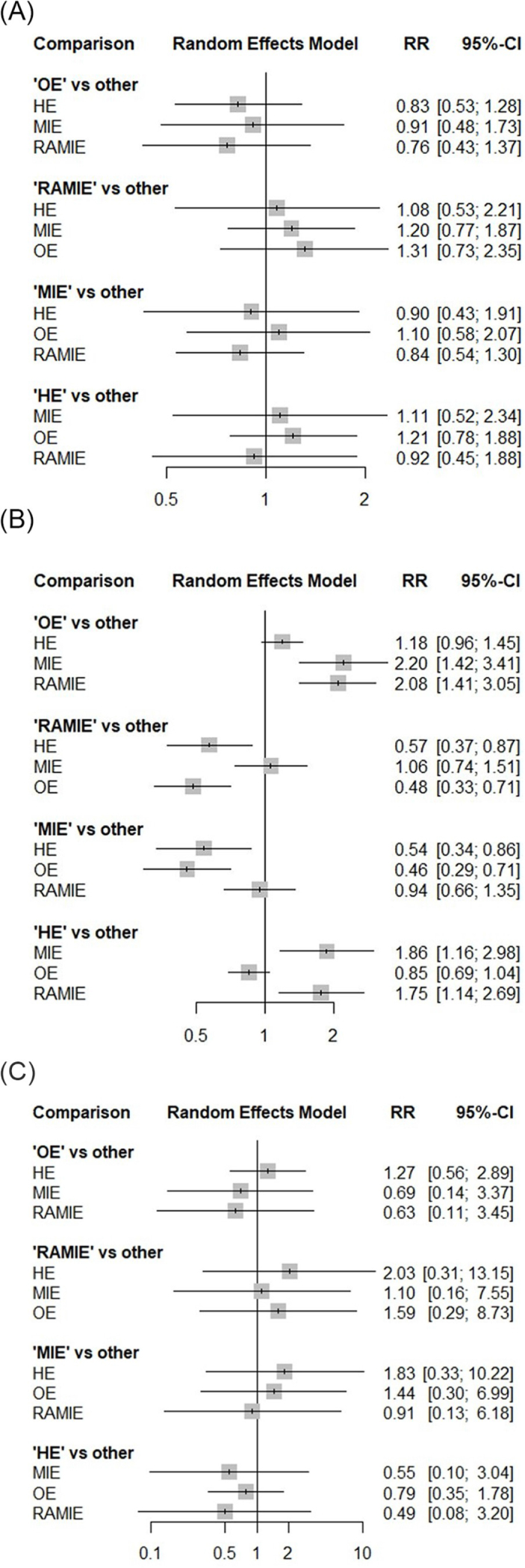
Network Forrest plot for anastomotic leak (A), pulmonary complications (B), and in-hospital mortality (C).

AL was reported in all RCTs (1772 patients)^[22,44–47,49,50,52]^. No significant differences were found for OE vs. HE (RR = 0.83; 95% CI 0.53–1.28), OE vs. MIE (RR = 0.91; 95% CI 0.48–1.73), and OE vs. RAMIE (RR = 0.76; 95% CI 0.43–1.37). Notably, no differences were found for MIE vs. RAMIE (RR = 0.84; 95% CI 0.54–1.30) and RAMIE vs. HE (RR = 1.08; 95% CI 0.53–2.21) (Fig. 2A). The treatment ranking plot indicated that OE (75%) had the highest likelihood of being the preferred treatment for low AL, followed by MIE (59%) and HE (39%) (Supplemental Digital Content Figure 2A, available at: http://links.lww.com/JS9/E619).

PCs was reported in all RCTs (1772 patients)^[22,44–47,49,50,52]^. MIE and RAMIE were associated with a reduced risk of PCs compared to OE (RR = 0.46; 95% CI 0.29–0.71 and RR = 0.48; 95% CI 0.33–0.71) and HE (RR = 0.54; 95% CI 0.34–0.86 and RR = 0.57; 95% CI 0.37–0.87). No differences were found for MIE vs. RAMIE (RR = 0.94; 95% CI 0.66–1.35) (Fig. 2B). The treatment ranking plot indicated that MIE (87%) had the highest likelihood of being the preferred treatment for low PCs, followed by RAMIE (79%), and HE (31%) (Supplemental Digital Content Figure 2B, available at: http://links.lww.com/JS9/E619).

**Figure 3.**
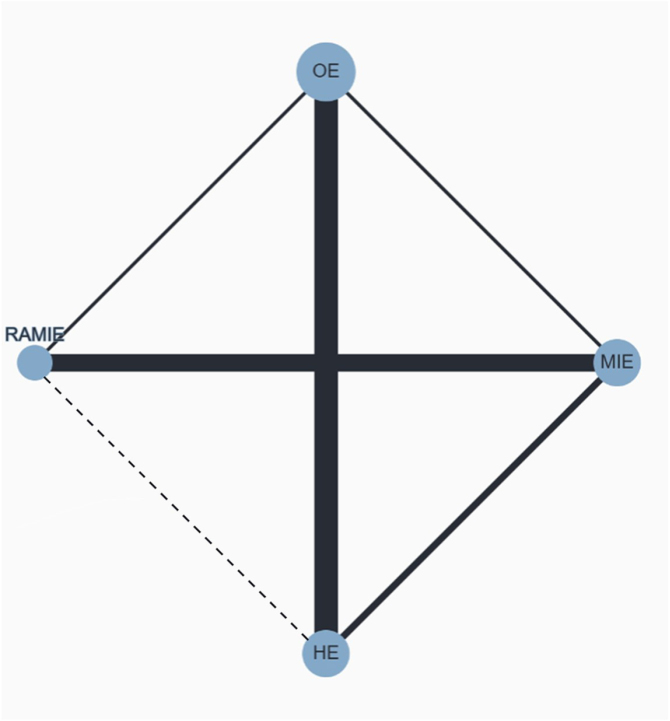
Network geometry for the primary outcomes. Nodes size reflects the sample size and edges width reflects the number of studies for a specific pairwise comparison. Solid line indicates direct comparisons while dotted line indicates indirect comparisons performed with the network methodology.

In-hospital mortality was reported in all RCTs (1772 patients)^[22,44–47,49,50,52]^. No significant differences were found for OE vs. HE (RR = 1.27; 95% CI 0.56–2.89), OE vs. MIE (RR = 0.69; 95% CI 0.14–3.37), and OE vs. RAMIE (RR = 0.63; 95% CI 0.11–3.45). Similarly, MIE vs. RAMIE (RR = 0.91; 95% CI 0.13–6.18) and RAMIE vs. HE (RR = 2.03; 95% CI 0.31–13.1) were comparable (Fig. 2C). The treatment ranking plot indicated that HE (57%) had the highest likelihood of being the preferred treatment for low in-hospital mortality, followed by OE (55%), and MIE (37%) (Supplemental Digital Content Figure 2C, available at: http://links.lww.com/JS9/E619). The network plot of all primary outcomes is depicted in Figure 3. The global heterogeneity was zero for all the primary outcomes while the prior sensitivity analysis yielded robust results for all treatment comparisons. The descriptive statistics, pooled network analysis, and heterogeneity for all primary outcomes are synthetized in Tables 2–3.

**Table 2:** Descriptive statistics stratified according to different treatment

OE		HE	MIE	RAMIE	
Categorical outcomes					
9.2 (6.8–20)		7.8 (1.8–21.4)	7.6 (0.9–11.9)	13.9 (11.6–24.1)	Anastomotic leak
31.5 (25.4–41.6)		29.4 (21.6–42.8)	17.3 (10.2–30)	18.3 (12.1–26.2)	Clavien-Dindo >2
6.9 (3.8–21.8)		4.4 (4.3–4.9)	1.8 (1.1–3)	6.8 (0.9–31.5)	Chylothorax
2.7 (0.0–3.6)		1.1 (0.4–7.1)	NA	1.8 (1.8–1.8)	Gastric conduit necrosis
1.8 (1.5–8.3)		1.5 (0.0–1.6)	0.6 (0.0–3.4)	0.5 (0.0–3.7)	In-hospital mortality
1.7 (0.0–2.3)		1.4 (0.9–1.5)	0.4 (0.0–1.7)	0.3 (0.0–1.8)	30-day mortality
2.7 (1.5–8.3)		1.6 (0.0–3.9)	0.6 (0.0–3.5)	0.6 (0.0–3.7)	90-day mortality
37.4 (25–58)		23.9 (8.2–33.3)	11.2 (2.7–14.7)	12.6 (3.1–15.9)	Pulmonary complications
81.9 (69.2–100)		81.7 (76.1–95.1)	97.2 (96.4–98.2)	94.9 (94.2–96.1)	R0
17.4 (10.7–33)		17.6 (12.7–28.6)	13.5 (13.5–13.5)	24.1 (24.1–24.1)	Return to OR
9.3 (5.8–14.3)		3.3 (0.9–5.9)	18.3 (0.0–33)	24.8 (9.3–32.6)	Vocal cords paralysis
6.4 (1.9–14.5)		3.6 (0.9–4.6)	0.5 (0.4–0.6)	2.1 (1.7–3.7)	SSI
Continuous outcomes					
312 (290–330)	272 (219–327)		282 (245–333)	270 (203–349)	Operative time (min)
521 (475–568)	590 (590–590)		193 (150–219)	201 (100–400)	Intraoperative blood loss (ml)
24.4 (21–26)	22.2 (19.2–24)		23.5 (20–25)	25.4 (23–29)	Total number of lymph nodes (n)
12.1 (10–16)	11.8 (11–14)		9.8 (9–11)	10.1 (9–14)	HLOS (days)
1.3 (1–4)	3.4 (3.2–5)		1.6 (1–3.3)	1 (1–1)	ICU-LOS (days)

Values are presented as percentages for categorical variables and as mean (range) for continuous variables.

HE, hybrid esophagectomy; OE, open esophagectomy; MIE, minimally invasive esophagectomy; RAMIE, robotic-assisted esophagectomy; NA, not available.

**Table 3:** League table

Categorical variables				*I*^2^	No. RCTs (no. patients)	Outcomes
OE	1.21 (0.78–1.88)	1.10 (0.58–2.07)	1.31 (0.73–2.35)	0.0	8 (1772)^[22,43–46,48,49,51]^	Anastomotic leak
0.83 (0.53–1.28)	HE	0.90 (0.43–1.91)	1.08 (0.53–2.21)			
0.91 (0.48–1.73)	1.11 (0.52–2.34)	MIE	1.20 (0.77–1.87)			
0.76 (043–1.37)	0.92 (0.45–1.88)	0.84 (0.54–1.30)	RAMIE			
OE	0.91 (0.74–1.12)	0.97 (0.47–2.01)	0.95 (0.50–1.80)	0.0	6 (1420 pts)^[22,43–46,48]^	Clavien-Dindo >2
1.10 (0.89–1.36)	HE	1.06 (0.50–2.27)	1.04 (0.53–2.04)			
1.03 (0.50–2.14)	0.94 (0.44–2.01)	MIE	0.98 (0.69–1.39)			
1.05 (0.56–2.00)	0.96 (0.49–1.88)	1.02 (0.72–1.45)	RAMIE			
OE	0.95 (0.48–1.89)	1.24 (0.31–4.93)	1.43 (0.71–2.86)	6.6	5 (1394 pts)^[22,43–46]^	Chylothorax
1.05 (0.53–2.10)	HE	1.31 (0.28–6.11)	1.50 (0.56–3.99)			
0.80 (0.20–3.18)	0.76 (0.16–3.55)	MIE	1.15 (0.35–3.76)			
0.70 (0.35–1.41)	0.67 (0.25–1.77)	0.87 (0.27–2.86)	RAMIE			
OE	0.52 (0.16–1.74)	NA	0.61 (0.07–5.19)	13.3	4 (859 pts)^[22,44,46,48]^	Gastric conduit necrosis
1.92 (0.57–6.43)	HE	NA	1.17 (0.10–13.70)			
NA	NA	MIE	NA			
1.64 (0.19–13.91)	0.85 (0.07–9.95)	NA	RAMIE			
OE	0.65 (0.33–1.28)	2.91 (0.34–24.89)	2.75 (0.53–14.14)	0.0	6 (1331 pts)^[22,43–46,51]^	90-day mortality
1.53 (0.78–2.99)	HE	4.45 (0.50–39.71)	4.20 (0.74–23.78)			
0.34 (0.04–2.94)	0.22 (0.03–2.00)	MIE	0.94 (0.17–5.14)			
0.36 (0.07–1.87)	0.24 (0.04–1.35)	1.06 (0.19–5.77)	RAMIE			
OE	0.67 (0.24–1.87)	3.81 (0.31–46.16)	2.29 (0.19–27.74)	0.0	6 (1512 pts)^[22,43–46,49]^	30-day mortality
1.49 (0.53–4.14)	HE	5.66 (0.38–83.98)	3.40 (0.23–50.47)			
0.26 (0.02–3.19)	0.18 (0.01–2.62)	MIE	0.60 (0.07–5.25)			
0.44 (0.04–5.30)	0.29 (0.02–4.36)	1.66 (0.19–14.53)	RAMIE			
OE	0.79 (0.35–1.78)	1.44 (0.30–6.99)	1.59 (0.29–8.73)	0.0	8 (1772)^[22,43–46,48,49,51]^	In-hospital mortality
1.27 (0.56–2.89)	HE	1.83 (0.33–10.22)	2.03 (0.31–13.15)			
0.69 (0.14–3.37)	0.55 (0.10–3.04)	MIE	1.10 (0.16–7.55)			
0.63 (0.11–3.45)	0.49 (0.08–3.20)	0.91 (0.13–6.18)	RAMIE			
OE	0.85 (0.69–1.04)	0.46 (0.29–0.71)	0.48 (0.33–0.71)	0.0	8 (1772)^[22,43–46,48,49,51]^	Pulmonary complications
1.18 (0.96–1.45)	HE	0.54 (0.34–0.86)	0.57 (0.37–0.87)			
2.20 (1.42–3.41)	1.86 (1.16–2.98)	MIE	1.06 (0.74–1.51)			
2.08 (1.41–3.05)	1.75 (1.14–2.69)	0.94 (0.66–1.35)	RAMIE			
OE	0.99 (0.93–1.06)	1.05 (0.97–1.14)	1.02 (0.94–1.10)	44.3	7 (1492 pts)^[22,43–46,48,49]^	R0
1.01 (0.94–1.08)	HE	1.06 (0.96–1.18)	1.03 (0.93–1.14)			
0.95 (0.87–1.03)	0.94 (0.85–1.05)	MIE	0.97 (0.92–1.02)			
0.98 (0.91–1.06)	0.97 (0.88–1.08)	1.03 (0.98–1.09)	RAMIE			
OE	1.08 (0.79–1.47)	1.24 (0.48–3.22)	0.74 (0.41–1.35)	0.0	5 (974 pts)^[22,44,46,48,49]^	Return to OR
0.93 (0.68–1.27)	HE	1.15 (0.42–3.14)	0.69 (0.35–1.35)			
0.81 (0.31–2.09)	0.87 (0.32–2.37)	MIE	0.60 (0.19–1.84)			
1.35 (0.74–2.44)	1.45 (0.74–2.83)	1.67 (0.54–5.14)	RAMIE			
OE	1.06 (0.29–3.83)	0.47 (0.15–1.48)	0.47 (0.16–1.43)	64.6	6 (1211 pts)^[22,43,45,46,49,51]^	Vocal cords paralysis
0.94 (0.26–3.39)	HE	0.44 (0.09–2.21)	0.44 (0.09–2.20)			
2.12 (0.67–6.64)	2.25 (0.45–11.16)	MIE	1.00 (0.52–1.93)			
2.11 (0.70–6.39)	2.25 (0.45–11.13)	1.00 (0.52–1.93)	RAMIE			
OE	0.71 (0.36–1.38)	0.13 (0.01–1.36)	0.30 (0.08–1.17)	0.0	4 (1191 pts)^[22,44–46]^	SSI
1.41 (0.73–2.74)	HE	NA	0.42 (0.09–1.92)			
7.62 (0.74–78.90)	5.40 (0.48–61.36)	MIE	2.28 (0.34–15.28)			
3.34 (0.86–13.00)	2.37 (0.52–10.75)	NA	RAMIE			
**Continuous variables**						
OE	−0.01 (−0.65, 0.65)	0.70 (−0.04, 1.36)	0.62 (−0.11, 1.35)	89.4	7 (1239 pts)^[22,43,45,46,48,49,51]^	Operative time^a^
0.01 (−0.65, 0.65)	HE	0.71 (−0.02, 1.43)	0.62 (−0.23, 1.48)			
−0.70 (−1.36, 0.04)	−0.71 (−1.43, 0.02)	MIE	−0.08 (−0.69, 0.52)			
−0.62 (−1.35, 0.11)	−0.62 (−1.48, 0.23)	0.08 (−0.52, 0.69)	RAMIE			
OE	0.93 (−0.15, 1.71)	−0.46 (−0.95, −0.04)	−0.69 (−1.19, −0.19)	77.4	5 (1006 pts)^[22,43,45,49,51]^	Intraoperative blood loss^a^
−0.93 (−1.71, 0.15)	HE	−1.39 (−1.99; −0.78)	−1.62 (−2.33, −0.91)			
0.46 (0.04, 0.95)	1.39 (0.78, 1.99)	MIE	−0.23 (−0.14, 0.61)			
0.69 (0.19, 1.19)	1.62 (0.91, 2.33)	0.23 (−0.14, 0.61)	RAMIE			
OE	−0.19 (−0.34, 0.04)	0.04 (−0.17, 0.25)	0.16 (−0.08, 0.40)	9.8	7 (1708 pts)^[22,43–46,49,51]^	Total number of lymph nodes^a^
0.19 (−0.04, 0.34)	HE	0.23 (−0.02, 0.44)	0.35 (−0.10, 0.60)			
−0.04 (−0.25, 0.17)	−0.23 (−0.44, 0.02)	MIE	0.12 (−0.04, 0.29)			
−0.16 (−0.08, 0.40)	−0.35 (−0.60, 0.10)	−0.12 (−0.29, 0.04)	RAMIE			
OE	0.24 (−0.03, 0.50)	−0.33 (−0.65, −0.01)	−0.38 (−0.74, −0.03)	62.2	8 (1772)^[22,43–46,48,49,51]^	HLOS^a^
−0.24 (−0.50, 0.03)	HE	−0.57 (−0.91, −0.23)	−0.62 (−1.01, −0.23)			
0.33 (0.01, 0.65)	0.57 (0.23, 0.91)	MIE	−0.05 (−0.34, 0.23)			
0.38 (0.03, 0.74)	0.62 (0.23, 1.01)	0.05 (−0.23, 0.34)	RAMIE			
OE	−0.11 (−0.45, 0.23)	0.02 (−0.24, 0.28)	0.02 (−0.25, 0.28)	0.0	6 (1032 pts)^[22,43,45,48,49,51]^	ICU-LOS^a^
0.11 (−0.23, 0.45)	HE	0.13 (−0.12, 0.38)	0.13 (−0.16, 0.42)			
−0.02 (−0.28, 0.24)	−0.13 (−0.38, 0.12)	MIE	0.00 (−0.16, 0.15)			
−0.02 (−0.28, 0.25)	−0.13 (−0.42, 0.16)	0.00 (−0.15, 0.16)	RAMIE			

Values are expressed as risk ratio (RR) and 95% confidence intervals (95% CI). *I*^2^ heterogeneity. Values are expressed as risk ratio (RR) and 95% CI.

^a^ Weighted mean differences.

### Meta-analysis – secondary outcomes

Data on conversion to open, reintubation, ICU readmission, hospital readmission after discharge and patients’ quality of life were not computed in the quantitative synthesis because the paucity of studies reporting these outcomes. All the other secondary outcomes are presented in Tables 2 and 3.

## Discussion

This study shows that compared to OE and HE, MIE and RAMIE were associated with significantly reduced postoperative PCs, intraoperative blood loss, and HLOS. AL, severe postoperative complications, chylothorax, vocal cord paralysis, GCN, reoperation, SSI, R0, total number of retrieved lymph nodes, OT, and in hospital mortality were comparable among treatments. MIE and RAMIE were comparable in term of perioperative outcomes and surgical radicality.

Over the past 20 years, significant advancements have been made in the management of esophageal cancer. The current NCCN and ESMO guidelines recommend using a transthoracic route to enhance mediastinal lymphadenectomy however, the best transthoracic approach is still not defined^[53,54]^. Since its adoption in clinical practice, transthoracic MIE has been shown to be comparable to OE in terms of safety and oncological outcomes. The TIME and MIRO trial demonstrated substantial improvement for MIE and HE in terms of reduced major postoperative and pulmonary complications compared to OE. However, technical limitations and poor ergonomics have posed challenges for radical oncological dissection particularly along the recurrent laryngeal nerves and anastomosis formation^[17]^. The introduction of robotic platforms has renewed enthusiasm due to improved ergonomics and high-definition 3D visualization. Additionally, the enhanced range of motion of robotic instruments has improved dissection at the thoracic apex for bilateral recurrent nerve lymphadenectomy, and easier anastomosis formation^[21,55]^. However, in anatomically challenging situations (i.e. cT4b tumors, enlarged paratracheal or subaortic lymph nodes, adhesions from previous surgeries, radiotherapy, or complex salvage procedures), OE and HE still remains essentials in the esophageal surgeon’s armamentarium^[14,56]^. Despite the recent advancements in perioperative and intensive care management, in-hospital mortality rates remain up to 3% in referral centers. In the present study, the estimated postoperative in-hospital mortality rates for OE, HE, MIE, and RAMIE were 2%, 1%, 0.4%, and 0.6%, respectively. The quantitative analysis revealed no significant differences among OE, HE, MIE, and RAMIE. Although the zero percent heterogeneity (*I*^2^ = 0.0%), our findings mandate cautions because possibly influenced by several factors such as different postoperative critical care strategies, operating surgeon’s learning curve, hospital volume, and non-defined individual patient causes of death.

The esophagogastric anastomosis is a critical component of esophagectomy and can significantly impact both short – and long-term morbidity and mortality. AL is associated with increased postoperative morbidity, higher mortality rates, prolonged hospital stays, elevated healthcare costs, reduced long-term quality of life and worse oncological survival^[57]^. Several factors may contribute to anastomotic failure, including the absence of a serosa layer, tension, inadequate blood supply to the gastric conduit, ischemic conditioning, surgical technique (stapled vs. hand-sewn and circular vs. linear stapled), malnutrition, and patient comorbidities^[58]^. In current literature, the incidence of AL ranges from 10% to 20%. In our study, the estimated incidence of AL in OE, HE, MIE, and RAMIE was 9%, 7.8%, 7.6%, and 13.9%, respectively. Notably, no significant differences were found in the quantitative network analysis among treatments thus indicating a limited effect of the surgical approach on AL. Our findings align with those reported by Sihag *et al*^[59]^ in their 2016 retrospective national dataset study, which included over 2700 patients and found no significant differences in AL rates between OE and MIE (6.9% vs. 8.1%; *P* = 0.3). Also, the TIME trial (7% vs. 12%; *P* = 0.39), the MIRO trial (7% vs. 11%), and the RAMIE trial (12.2% vs. 11.3%; *P* = 0.81) concluded no differences in postoperative AL rates when comparing OE vs. MIE, OE vs. HE, and RAMIE vs. MIE^[46,47,50]^. Similarly, Patton *et al*^[27]^, in their meta-analysis reported similar AL odds for OE, HE, MIE, and RAMIE. In contrast, Angeramo *et al*^[60]^ concluded that OE had a significantly reduced AL OR compared to MIE (OR = 0.71; *P* < 0.001). PCs after esophagectomy are commonly reported, with an incidence rate up to 30%. These complications are associated with extended hospital stays, higher healthcare costs, increased perioperative mortality, and reduced long-term survival rates^[61,62]^. In our study, the incidence of PCs in OE, HE, MIE, and RAMIE was 37%, 23%, 11%, and 12%, respectively. The quantitative analysis revealed a significantly reduced risk of PCs for MIE and RAMIE compared to OE and HE. This result may be attributed to the reduction in surgical/diaphragmatic trauma, improved visualization and preservation of vagal pulmonary branches, less postoperative pain and a lower incidence of diaphragmatic splinting with reduced basal lung atelectasis. Further, it has been postulated that the bilateral lung ventilation in prone or semi prone position used in MIE and RAMIE is associated with minimized arteriovenous shunt, improved ventilation-perfusion match, minimized ischemia-reperfusion injury/oxidative stress, and better intraoperative oxygenation^[63]^. Our findings align with the TIME trial, which reported a higher rate of in-hospital PCs for OE vs. MIE (31% vs. 12%; *P* = 0.005)^[50]^. Similarly, our results corroborate the conclusions of the MIRO trial that conveyed a higher incidence of postoperative PCs in OE vs. HE (30% vs. 18%)^[47]^. Interestingly, our results for MIE vs. RAMIE showed equivalent results (RR = 0.94; 95% CI 0.66–1.35) and were consistent with those reported in the REVATE and RAMIE trials^[44,46]^. The robustness of these primary results was confirmed by the related zero percent heterogeneity. While interpreting our results caution is mandatory because the presence of possible confounders such as: different patient baseline comorbidities (i.e. smoking status, peripheral vascular disease, coronary artery disease, celiac trunk calcification, etc.), anastomotic technique (hand-sewn vs. stapled; circular stapled vs. linear stapled)^[64]^, anastomosis location (intrathoracic vs. cervical), ICG utilization, ischemic conditioning strategies, surgeons’ experience, learning curves, and hospital volume. Further, the different definitions of PCs, detection bias, antibiotics protocols, ERAS pathways, epidural anaesthesia administration, prehabilitation and/or rehabilitations programs, and the ventilation technique (single lung ventilation vs. dual lung ventilation vs. single lung ventilation with bronchial blocker) should be pondered.

Notably, MIE and RAMIE demonstrated a reduction of HLOS compared to OE and HE. These findings may be attributed to reduced tissue trauma, decreased postoperative pain, fewer pulmonary complications, and a lower systemic stress response. Further, MIE and RAMIE also show a trend toward decreased intraoperative blood loss probably reflecting the reduced surgical trauma and limited incision length. No differences in postoperative vocal cord paralysis and chylothorax were observed across the treatments. Although these findings may seem unexpected considering the more precise tissue dissection in MIE and RAMIE, it’s important to consider the substantial influence of surgeon expertise, the variability in lymphadenectomy along the recurrent nerves, as well as the strategies for ligation, resection, or preservation of the thoracic duct^[61,65–67]^. The R0 rates and the average number of harvested lymph nodes were comparable across treatments thus emphasising the oncological validity of all approaches. The RAMIE and REVATE trials previously suggested that RAMIE may be more effective during thoracic lymphadenectomy, especially around the left recurrent laryngeal nerve, resulting in a greater number of harvested lymph nodes^[44,46,68]^. Since this outcome was not detailed in the other trials, a comprehensive quantitative analysis could not be performed. Future studies seem important to investigate this issue, considering that lymph node metastasis along the recurrent laryngeal nerve is a substantial prognostic indicator.

The performance of surgeons, proficiency, learning curves, structured training and mentorship programs, and the volume of cases handled by hospitals can significantly influence patient outcomes and introduce bias. Research has highlighted that these operator-related factors are crucial in determining OT, intraoperative blood loss, the total number of harvested lymph nodes, complications, and mortality rates. It has been reported that to achieve a low incidence of AL (<8%) in the context of MIE, over 100 cases must be performed to attain proficiency^[69]^. It’s essential to recognize that the short-term outcomes evaluated in the present network analysis, may not only reflect the impact of a specific surgical technique but may also depend on the surgeon’s experience and the hospital’s case volume.

The study was conducted in accordance with PRISMA guidelines and adhered to a rigorous methodology outlined in the PROSPERO protocol from the outset^[28]^. This encompassed comprehensive outcome measures and assessments of quality at both the study level (risk of bias) and the outcome level (CINeMA)^[35,41]^. The selection criteria resulted in a homogeneous population for primary outcomes, as indicated by low heterogeneity. By employing network meta-analytical techniques, we were able to synthesize data globally, rank the treatments, and conduct both direct and indirect comparisons. Notably, the indirect comparison facilitated by the current network geometry proved valuable for preliminarily assessing the comparison between RAMIE and HE, which has not been previously explored in the context of RCTs. The present study has some limitations. First, while the transitivity assumption was upheld with no significant statistical inconsistency in the network analysis, the accuracy of our findings may be influenced by variations in the proficiency of operating surgeons, which could affect postoperative complications. Second, certain confounders, such as the lack of a consistent surgical approach (Ivor Lewis and McKeown), anastomosis locations (cervical and intrathoracic), and the absence of standardized postoperative management protocols, may affect some of the outcomes considered. Third, the anastomotic techniques varied based on the preferences of individual surgeons. Fourth, the definitions and reporting of outcomes (detection bias) may differ among the included studies, although it can be assumed that these inconsistencies would be evenly spread across treatment groups. This aspect further emphasizes the necessity for standardized reporting of postoperative outcomes (ECCG classification)^[70]^. Fifth, the impact of novel neoadjuvant treatments/protocols on postoperative short-term outcomes remains unclear and requires further clarification^[5]^. Lastly, data concerning costs, patient-reported outcomes, and long-term survival were not analysed and require future research.

## Conclusions

This study shows that OE, HE, MIE, and RAMIE have equivalent safety profiles. MIE and RAMIE were associated with significantly reduced postoperative PCs, intraoperative blood loss, and HLOS compared to OE and HE whereas no differences were observed for the other short-term outcomes among treatments. MIE and RAMIE showed equivalent perioperative outcomes and oncological radicality.

## Data Availability

All data generated or analyzed during this study are included in this published article [and its supplementary information files].

## Research registration unique identifying number (UIN)

The protocol for this meta-analysis has been registered with PROSPERO (CRD42025645818).
